# ACTH treatment promotes murine cardiac allograft acceptance

**DOI:** 10.1172/jci.insight.143385

**Published:** 2021-07-08

**Authors:** Jing Zhao, Liwei Jiang, Mayuko Uehara, Naima Banouni, Basmah S. Al Dulaijan, Jamil Azzi, Takaharu Ichimura, Xiaofei Li, Petr Jarolim, Paolo Fiorina, Stefan G. Tullius, Joren C. Madsen, Vivek Kasinath, Reza Abdi

**Affiliations:** 1 Transplantation Research Center,; 2 Renal Division, and; 3 Department of Pathology, Brigham and Women’s Hospital, Harvard Medical School, Boston, Massachusetts, USA.; 4 Department of Nephrology, Boston Children’s Hospital, Harvard Medical School, Boston, Massachusetts, USA.; 5 International Center for Type 1 Diabetes, Centro di Ricerca Pediatrica Romeo ed Enrica Invernizzi, Dipartimento di Scienze Biomediche e Cliniche “L. Sacco”, Università di Milano, Milan, Italy.; 6 Endocrinology Division, ASST Fatebenefratelli Sacco, Milan, Italy.; 7 Division of Transplant Surgery, Department of Surgery, Brigham and Women’s Hospital, Harvard Medical School, Boston, Massachusetts, USA.; 8 Center for Transplantation Sciences, Department of Surgery, and; 9 Division of Cardiac Surgery, Department of Surgery, Massachusetts General Hospital and Harvard Medical School, Boston, Massachusetts, USA.

**Keywords:** Immunology, Transplantation, Organ transplantation, T cell receptor, Tolerance

## Abstract

Heart transplantation is the optimal therapy for patients with end-stage heart disease, but its long-term outcome remains inadequate. Recent studies have highlighted the importance of the melanocortin receptors (MCRs) in inflammation, but how MCRs regulate the balance between alloreactive T cells and Tregs, and whether they impact chronic heart transplant rejection, is unknown. Here, we found that Tregs express MC2R, and MC2R expression was highest among all MCRs by Tregs. Our data indicate that adrenocorticotropic hormone (ACTH), the sole ligand for MC2R, promoted the formation of Tregs by increasing the expression of IL-2Rα (CD25) in CD4^+^ T cells and activation of STAT5 in CD4^+^CD25^+^ T cells. ACTH treatment also improved the survival of heart allografts and increased the formation of Tregs in CD28KO mice. ACTH treatment synergized with the tolerogenic effect of CTLA-4–Ig, resulting in long-term survival of heart allografts and an increase in intragraft Tregs. ACTH administration also demonstrated higher prolongation of heart allograft survival in transgenic mouse recipients with both complete KO and conditional KO of PI3Kγ in T cells. Finally, ACTH treatment reduced chronic rejection markedly. These data demonstrate that ACTH treatment improved heart transplant outcomes, and this effect correlated with an increase in Tregs.

## Graphical abstract

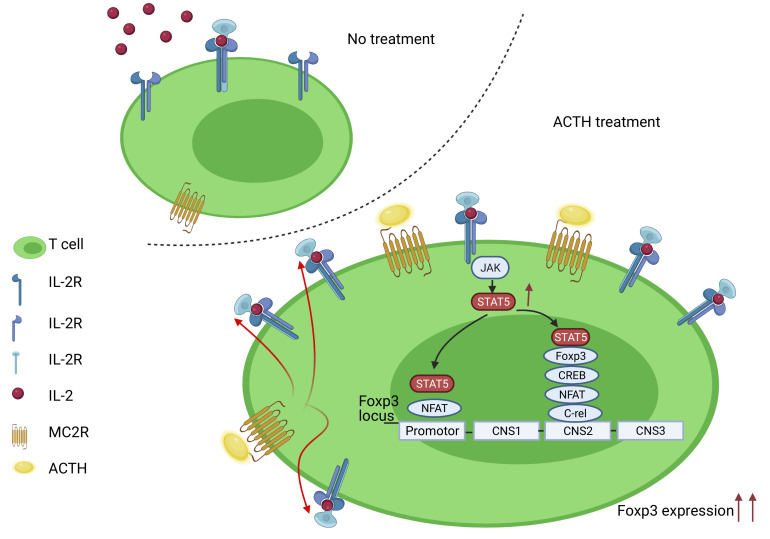

## Introduction

Heart transplantation is a life-saving strategy; however, numerous challenges must be met to improve allograft and recipient survival (1, 2). An average of 4000 heart transplants are performed in the United States annually. Despite an improvement in short-term outcomes, a high percentage of heart transplant recipients develop chronic rejection (3, 4).

Cardiac allograft vasculopathy (CAV) refers to the development of diffuse coronary artery disease of the allograft that rises in incidence 1 year following transplantation and represents an important sequela of excessive inflammation related to graft rejection (5, 6). CAV is a typical cause of graft dysfunction, affecting about 30% of patients at 5 years after transplantation (5). Besides malignancy, CAV causes the most deaths in heart transplant recipients after the third year after transplantation (5). Moderate or severe coronary artery disease that is diagnosed within 1 year after transplantation, termed early CAV, is an independent predictor of a mortality rate of 50% at 3 years after transplantation (7). CAV also persists as a major hindrance against long-term survival of the transplant (7).

In addition to lifelong maintenance immunosuppressive therapy, the adoption of induction therapies (i.e., T cell–depleting agents) by the majority of transplant programs has resulted in a marked decline in acute rejection rates. However, chronic rejection and graft loss rates remain virtually unchanged. More intense immunosuppression regimens have given rise to serious complications, including infection, malignancy, metabolic disorders, and microvascular toxicity (8–15). Most of the currently used immunosuppressive agents target both Tregs and effector T cells (Teffs) indiscriminately. Therefore, improvement of long-term heart transplant outcomes through the development of immune therapies that shift the Treg/Teff balance toward Tregs is a major unmet medical need (8, 12, 16–22).

The melanocortin receptor (MCR) family consists of a group of 7-transmembrane G-protein coupled receptors generally referred to as MC1R, MC2R, MC3R, MC4R, and MC5R (23). The importance of MCRs to the pathogenesis of inflammation has been recognized increasingly (24). The attachment of adrenocorticotropic hormone (ACTH) and α-, β-, and γ-melanocyte–stimulating hormones (α-, β-, and γ-MSH) to their cognate MCRs create a multitude of physiological responses (23, 25–27). However, the implications of MCR activity in the inflammatory response arose from the evident clinical utility of ACTH in treating inflammatory conditions, such as rheumatoid arthritis (28, 29). Most of these antiinflammatory properties were attributed initially to be glucocorticoid dependent, but later studies highlighted that ACTH and MSHs possess glucocorticoid-independent immunomodulatory effects by activating MCRs expressed by immune cells (30). Getting et al. revealed that ACTH exerted immunosuppressive effects in adrenalectomized rats with gouty arthritis of the knees, suggesting that these consequences existed independently of hypothalamus-pituitary-adrenal axis and glucocorticoid activity (24, 31). Acthar Gel has also demonstrated immunoregulatory function, such as in the treatment of acute exacerbations of multiple sclerosis in patients who have failed steroid therapy (32).

ACTH is the only ligand for MC2R, also known as the ACTH receptor (23, 33, 34). This property stands in contrast to MC1R, MC3R, MC4R, and MC5R, which are all bound by α-, β-, and γ-MSH, in addition to ACTH (35). MC2R is expressed by cells in the adrenal cortex, adipocytes, skin, pituitary gland, endometrium, erythroblasts, and osteoblasts (36), and its placement in the cell membrane relies uniquely among the MCRs on the expression of another molecule known as MCR accessory protein (MRAP; ref. 37). However, the expression of MC2R by lymphocytes is unknown, and its importance to determining the ratio between Teffs and Tregs remains to be fully studied.

Here, we investigated the expression of MCRs in various lymphocytes and tested the hypothesis that the interaction between ACTH and MC2R promotes the formation of Tregs and suppression of transplant rejection. We also examined synergism between ACTH and 2 emerging pathways of immunomodulation for cardiac transplantation — (a) costimulatory blockade with CTLA-4–Ig and (b) phosphoinositide 3-kinase-γ (PI3Kγ) absence — in extending heart allograft survival. CTLA-4–Ig has emerged as a promising suppressor of costimulation that can reduce or eliminate the need for calcineurin inhibitors (38–45). However, while CTLA-4–Ig decreases stimulation of Teffs, it also suppresses Tregs, thereby reducing its efficacy. The development of safer and more precisely targeted immunomodulatory treatment protocols that augment the regulatory axis while suppressing effector cells is a major unmet clinical need in transplantation. We found that the addition of ACTH has a synergistic effect with both CD28 inhibition and PI3Kγ absence in prolonging heart allograft survival. ACTH treatment also reduced the prevalence of CAV and chronic rejection in a mouse model of heart transplantation. Together, these findings have significant clinical implications, due to the impact of CAV on allograft dysfunction in heart transplant recipients and our identification of ACTH-MC2R as a molecular interaction that may exert significant antiinflammatory effects through the propagation of Tregs.

## Results

### ACTH promotes the formation of Tregs through MC2R.

**Figure 1:**
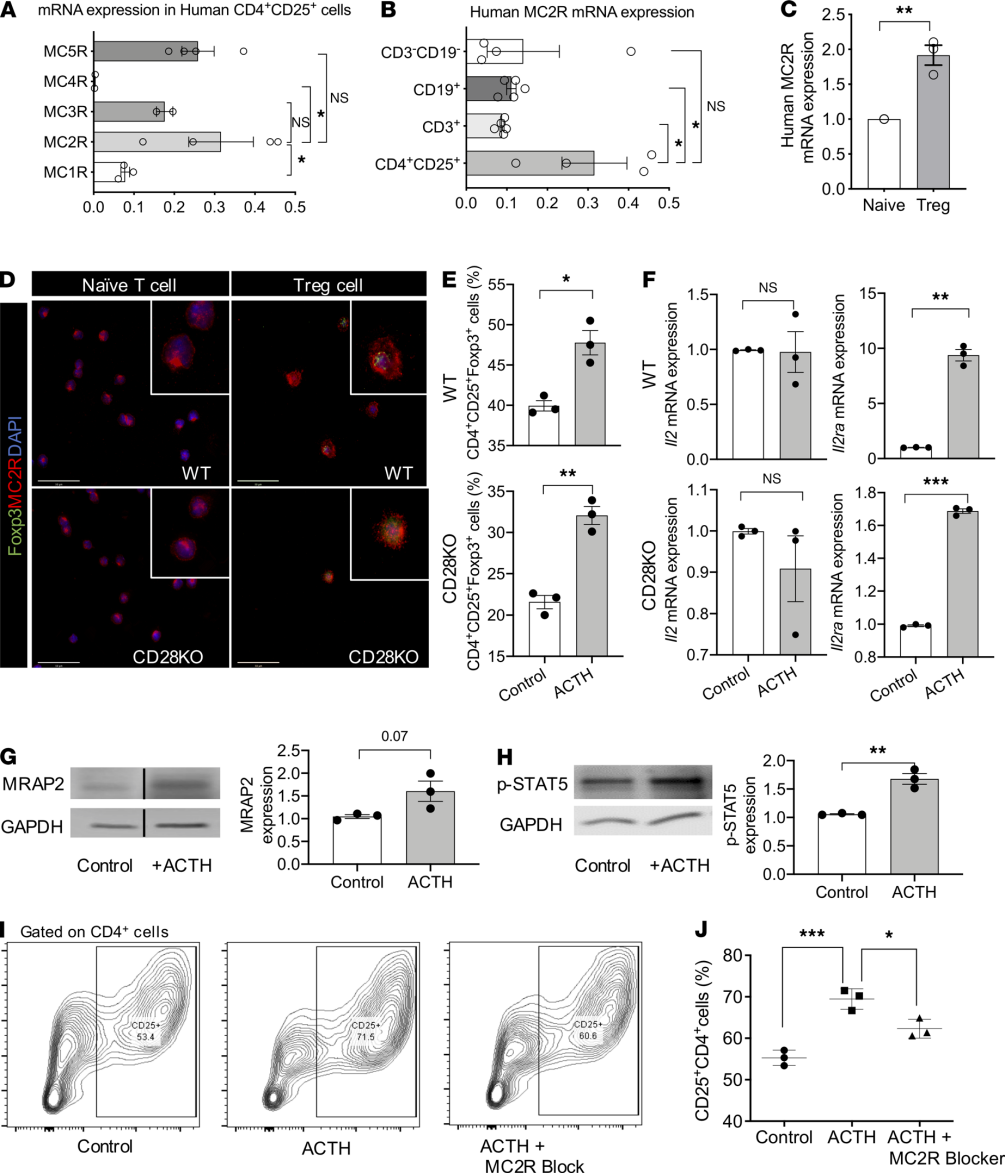
ACTH induces formation of Tregs through MC2R. (**A**) Comparison of MC1R, MC2R, MC4R, and MC5R gene expression by human CD4^+^CD25^+^ cells by qPCR (*n =* 3–5/group). (**B**) Comparison of MC2R gene expression by human CD3^–^CD19^–^ cells, CD19^+^ B cells, CD3^+^ T cells, and CD4^+^CD25^+^ T cells by qPCR (*n =* 3–5/group). (**C**) Comparison of MC2R gene expression by human Tregs versus naive T cells by qPCR (*n =* 3/group). (**D**) Immunofluorescence staining of MC2R expression in mouse Tregs and non-Tregs from WT and CD28KO mice. Scale bar: 50 μm. (**E**) Flow cytometric analysis of percentage of CD4^+^CD25^+^Foxp3^+^ cells (Tregs) in Treg induction assay of splenocytes from WT and CD28KO mice, following either treatment with ACTH or no treatment (Control). (**F**) Comparison of gene expression of *Il2ra* and *Il2* by Tregs of WT and CD28KO mice, following either ACTH treatment or no treatment (Control), by qPCR. (**G**) Comparison of MRAP2 protein expression by Tregs from CD28KO mice following ACTH treatment or no treatment (Control) by Western blot (*P =* 0.07). (**H**) Comparison of p-STAT5 gene expression by Tregs from CD28KO mice following ACTH treatment or no treatment (Control) by Western blot. (**I**) Representative flow cytometric plots of CD25 expression by CD4^+^ cells in Treg induction assay following treatment with ACTH and MC2R blocker. (**J**) Flow cytometric analysis of percentage of CD25^+^CD4^+^ cells following treatment with ACTH, ACTH, and MC2R blocker, or no treatment (Control) (*n =* 3/group). Data presented as mean ± SEM; *P < 0.05, **P < 0.01, ***P < 0.001 by 2-way ANOVA with Turkey’s multiple comparisons (**A**, **B**, and **J**) and Student’s *t* test (**C**, **E**–**H**).

We isolated lymphocytes from fresh human peripheral blood by MACS, and we separated CD3^+^ cells, CD19^+^ cells, CD4^+^CD25^+^ T cells, and CD3^–^CD19^–^ cells, as described above. Then, we measured the gene expression of MC1R, MC2R, MC3R, MC4R, and MC5R in the CD4^+^CD25^+^ T cells, and we found by quantitative PCR (qPCR) that MC2R was expressed more highly than the other MCRs (Figure 1A). Next, we compared MC2R gene expression by CD3^+^ cells, CD19^+^ cells, CD4^+^CD25^+^ T cells, and CD3^–^CD19^–^ cells, and we found that its expression was highest in the CD4^+^CD25^+^ T cells (Figure 1B). Meanwhile, we found that human CD4^+^CD25^+^Foxp3^+^ Tregs expressed MC2R significantly more robustly than naive T cells, as measured by qPCR (Figure 1C). In addition, expression of MC2R by WT mouse Tregs was higher than naive T cells by immunofluorescence staining (Figure 1D), reflecting a similar relationship between MC2R expression in CD28KO mouse Tregs and non-Tregs (Figure 1D).

To assess the effect of ACTH on Tregs, we first isolated lymphocytes from the spleens of WT and CD28KO mice. CD28 is required for Treg development, and IL-2 plays an important role in this process (46). We performed induction of Tregs in vitro using these cells. As shown in Figure 1E and Supplemental Figure 1A (supplemental material available online with this article; https://doi.org/10.1172/jci.insight.143385DS1), ACTH treatment resulted in a significantly higher percentage of CD4^+^CD25^+^Foxp3^+^ Tregs as a proportion of CD4^+^ cells in both WT and CD28KO mice. ACTH also increased the gene expression of *Foxp3*, the Treg transcription factor, significantly in CD28KO CD4^+^CD25^+^ T cells, as compared with an untreated control group, while no significant difference was observed in the WT CD4^+^CD25^+^ T cells (Supplemental Figure 1B). We also noted an increase in the expression of CD25, the IL-2 receptor α chain (IL-2Rα) (Supplemental Figure 1C), by CD4^+^ cells from both WT and CD28KO mice following treatment with ACTH; this effect was more prominent in the CD28KO splenocytes. CD25 is the IL-2R subunit with the highest affinity for IL-2, as compared with the β and γ chains that together comprise the tetrameric IL2αβγ receptor. Further analysis by qPCR revealed that ACTH treatment increased the expression of IL-2Rα, but it did not increase IL-2 expression by CD4^+^CD25^+^ T cells in both the WT and CD28KO mice (Figure 1F). Meanwhile, we checked the effect of ACTH on gene expression of other Treg promoters, such as *Nfatc2*, *Ap1*, *E2a*, and *Cre1*, by both WT and CD28KO mice, but we found no difference in these markers (Supplemental Figure 1B). We also found no significant difference between the IL-2 concentrations in media extracted from T cells cultured from the spleens of WT and CD28KO mice and treated with ACTH (Supplemental Figure 1D). Next, we performed suppression assays with ACTH to determine whether it exerts a direct effect on Teffs. As shown in Supplemental Figure 1E, no difference in the percentage of Teffs was observed between the untreated control and the ACTH-treated group.

Then, we investigated whether binding by ACTH leads to different effects in natural Tregs (nTreg) versus induced Tregs (iTreg) by isolating Tregs and non-Tregs from the spleens of WT mice and treating these cells with ACTH. As shown in Supplemental Figure 1F, flow cytometry demonstrated that ACTH did not significantly increase the percentage of nTregs, whereas the percentage of iTregs in a Treg induction assay was significantly higher following ACTH treatment in comparison with no treatment (Supplemental Figure 1G). In addition, no difference was observed between the ACTH-treated nTregs and the untreated nTregs in the percentage of proliferation (Ki-67^+^), apoptosis (Caspase 3), or expressions of the costimulatory molecules PD-1, TIM-3, LAG-3, and CTLA-4 (Supplemental Figure 1H).

Next, we investigated the effect of ACTH on the expression of the accessory protein MRAP2 by CD4^+^CD25^+^ T cells from the CD28KO mice. The presence of MRAP2 is necessary for insertion of MC2R in the cell membrane. We identified a trend toward stimulation of the expression of MRAP2 by ACTH in comparison with no treatment, with a *P* value that approached significance (*P =* 0.07; Figure 1G). We also examined the effect of ACTH on the expression of the phosphorylated signal transduction protein STAT5 (p-STAT5), a key molecule positioned downstream of IL-2R that promotes the expression of Foxp3 (47, 48). Addition of ACTH resulted in significantly higher expression of p-STAT5 expression by CD28KO CD4^+^CD25^+^ T cells (Figure 1H).

Next, we harvested PBMCs from human blood and isolated CD4^+^CD25^–^ T cells, as described above, to determine whether ACTH induces Tregs in human cells similarly to the induction in mouse cells. We found that treatment of these human PBMCs with ACTH increased human CD4^+^CD25^+^ T cells significantly, as determined by flow cytometry, while MC2R blocker blunted the induction of Tregs significantly (Figure 1, I and J), demonstrating that the differentiation of CD4^+^ T cells into Tregs induced by ACTH was mediated predominantly through the activity of MC2R.

### ACTH improves graft survival in CD28KO mice.

**Figure 2:**
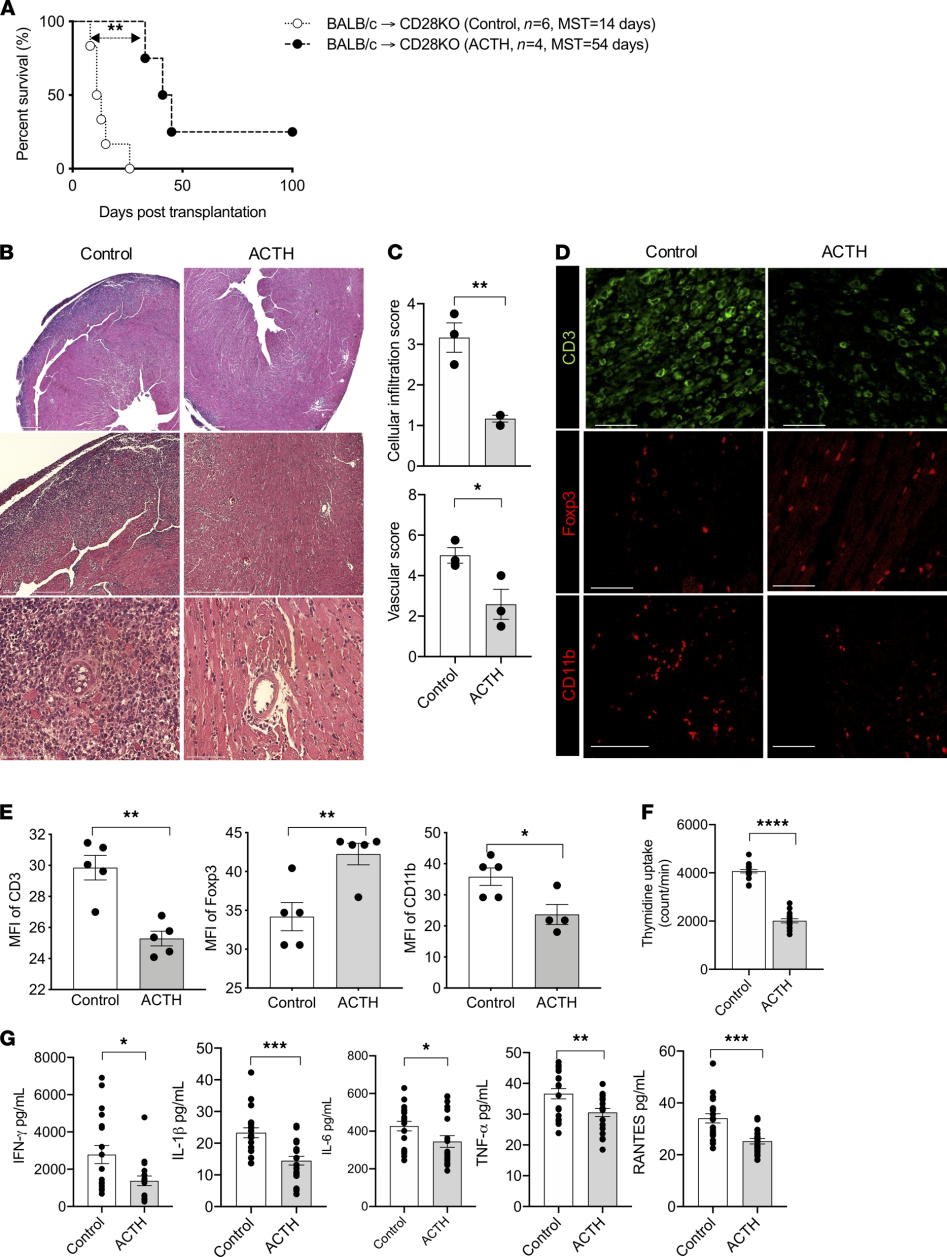
ACTH improves graft survival in CD28KO mice. (**A**) Comparison of heart allograft survival in CD28KO recipients of BALB/c hearts that received either ACTH (*n =* 4 mice/group; MST, 54 days) or no treatment (Control; *n =* 6 mice/group; MST, 14 days). **P < 0.01 by log-rank test. (**B**) Representative light micrographs of H&E-stained heart allograft sections from CD28KO recipients following either ACTH treatment or no treatment (Control) at day 7. Scale bars: 750 μm, 500 μm, and 75 μm (from top to bottom). (**C**) Comparison of cellular infiltration and vascular damage of the heart allografts in CD28KO recipients following either ACTH treatment or no treatment (Control). (**D**) Representative fluorescence micrographs of CD3^+^ T cell, CD11b^+^ cell, and Foxp3^+^ Treg populations in heart allograft sections following ACTH treatment or no treatment (Control: day 7 after transplant). Scale bars: 50 μm. (**E**) Bar graphs show comparison of MFIs of CD3, Foxp3, and CD11b staining in heart allograft sections following ACTH treatment or no treatment (Control: day 7 after transplant) (*n =* 3 mice/group). (**F**) T cell stimulation assay following ACTH treatment or no treatment. (**G**) Quantification of IFN-γ, IL-1β, IL-6, TNF-α, and RANTES concentrations following ACTH treatment or no treatment (Control) by ELIspot and Luminex assay. Data presented as mean ± SEM; *P < 0.05, **P < 0.01, ***P < 0.001 by Student’s *t* test.

A full MHC-mismatch model was used to assess the effect of ACTH on allograft survival. BALB/c mouse hearts were transplanted orthotopically into either WT or CD28KO recipient mice (BALB/c→C57BL/6; BALB/c→CD28KO). Notably, ACTH prolonged the survival of the heart allografts in the CD28KO mice (Figure 2A; MST, 14 days versus 54 days), but not in the WT mice (Supplemental Figure 2A; MST, 7 days versus 7.5 days). Analysis of heart allografts from these mice revealed moderate to severe cellular infiltration and occluded vasculature in the control group, while allografts from the ACTH-treated group contained much lower cellular infiltration and more intact vasculature (Figure 2, B and C). Immunofluorescence staining demonstrated that the heart allografts in the ACTH-treated group contained fewer CD3^+^ T cells and CD11b^+^ cells, along with a higher density of Foxp3^+^ cells, than the control group (Figure 2, D and E).

We were also interested in assessing the effect of ACTH on peripheral immune responses. Spleens and lymph nodes (LNs) were collected for flow cytometry. We observed no difference in the percentage of Tregs between the spleens and LNs of the 2 groups (Supplemental Figure 2, B and C). Nonetheless, the ratio between Tregs and Teffs, a surrogate measurement of the antiinflammatory nature of the peripheral immune microenvironment, was significantly higher in the spleens of mice treated with ACTH in comparison with the control group (Supplemental Figure 2C).

Next, we examined the effect of ACTH on alloreactivity using mixed lymphocyte reaction (MLR) and ELIspot assays. As shown in Figure 2F, ACTH suppressed the proliferation of T cells significantly in an MLR assay, in which irradiated splenocytes from BALB/c donors were used as stimulators and splenocytes from CD28KO mice were used as responders. Addition of ACTH also lowered the production of the proinflammatory cytokines IFN-γ, IL-1β, IL-6, TNF-α, and RANTES significantly (Figure 2G). These findings suggested that ACTH not only suppressed allograft rejection, but it also decreased activation of the systemic immune response, to an extent.

### Combination of ACTH with low-dose CTLA-4–Ig induces long-term graft survival.

**Figure 3:**
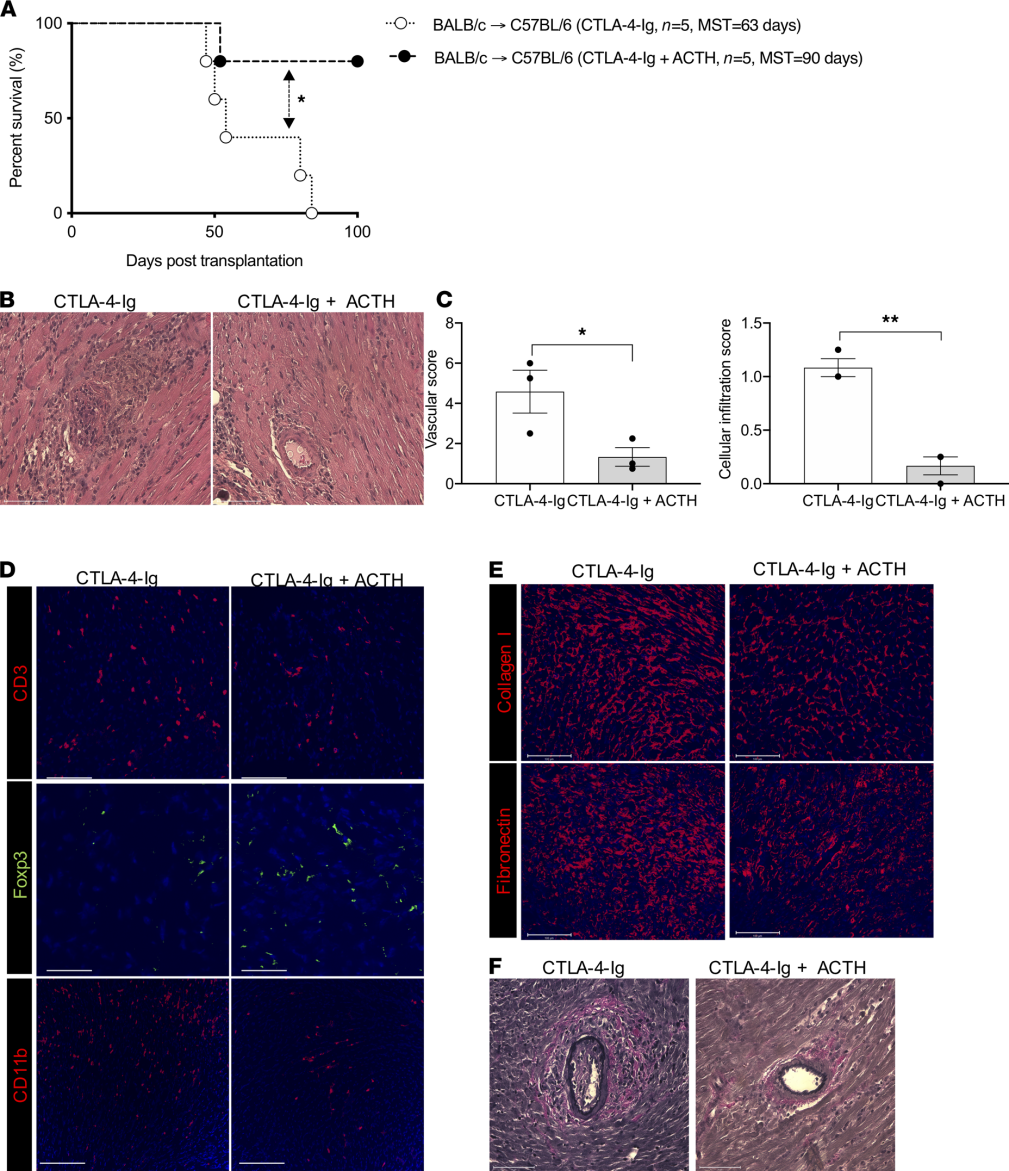
Combination of ACTH with low-dose CTLA-4–Ig induced long-term heart allograft survival. (**A**) Comparison of heart allograft survival between C57BL/6 recipients of BALB/c hearts that were treated with a combination of ACTH and CTLA-4–Ig (*n =* 5 mice/group; MST, 90 days) or CTLA-4–Ig alone (*n =* 5 mice/group; MST, 63 days). *P < 0.05 by log-rank test. (**B**) Representative light micrographs of H&E-stained heart allograft sections at day 28 from C57BL/6 recipients treated with a combination of ACTH and CTLA-4–Ig or CTLA-4–Ig alone. Scale bar: 75 μm. (**C**) Comparison of cellular infiltration and vascular damage of the heart allografts in CD28KO recipients following treatment with a combination of ACTH and CTLA-4–Ig or CTLA-4–Ig alone (*n =* 3 mice/group). (**D**) Representative florescence micrographs of CD3^+^ T cells, CD11b^+^ cells, and Foxp3^+^ Tregs in heart allograft sections of CD28KO recipients following treatment with a combination of ACTH and CTLA-4–Ig or CTLA-4–Ig alone. Scale bar: 100 μm. (**E**) Representative fluorescence micrographs of fibronectin and collagen I fibers in heart allograft sections of CD28KO recipients following treatment with a combination of ACTH and CTLA-4–Ig or CTLA-4–Ig alone. Scale bar: 100 μm. (**F**) Light micrographs of Verhoeff’s-stained heart allograft sections of CD28KO recipients following treatment with a combination of ACTH and CTLA-4–Ig or CTLA-4–Ig alone. Scale bar: 75 μm. Data presented as mean ± SEM; *P < 0.05, **P < 0.01 by Student’s *t* test.

After determining that ACTH lengthens allograft survival in CD28KO recipient mice, we wanted to test whether it synergized with CTLA-4–Ig to extend this prolongation. CTLA-4–Ig is a competitive inhibitor of the CD28/B7 pathway and has been used as an immunosuppressive agent. Costimulation blockade through the use of CTLA-4–Ig has emerged as a potential means for immunosuppression maintenance following transplantation in humans, sparing the classic metabolic and microvascular toxicity associated with calcineurin inhibitors (CNIs). CTLA-4–Ig inhibits Teffs, but previous studies have demonstrated that it also diminishes the Treg population simultaneously through CD28 blockade, likely dulling its efficacy in preventing transplant rejection (49–55). Hearts from BALB/c mice were transplanted into C57BL/6 recipients that were treated with either CTLA-4–Ig alone or CTLA-4–Ig + ACTH. ACTH synergized with the immunosuppressive effect conferred by CTLA-4–Ig and extended the mean survival of the heart allografts by at least 27 days in comparison with the mice that received CTLA-4–Ig alone (Figure 3A).

In addition, heart allografts from another group of mice in which the above experiment was repeated were harvested at day 28 to assess chronic transplant rejection. H&E staining revealed moderate to severe injury of the heart allograft, with more severe cellular infiltration and occluded vasculature in the group treated with CTLA-4–Ig alone, as compared with the group treated with CTLA-4–Ig combined with ACTH (Figure 3B). Pathologic scoring revealed significantly lower cellular and vascular injury in the CTLA-4–Ig + ACTH group (Figure 3C). Immunofluorescence staining of heart allografts revealed fewer CD3^+^ T cells and CD11b^+^ cells, but more Foxp3^+^ Tregs, in the CTLA-4–Ig + ACTH treatment group, as compared with the CTLA-4–Ig group (Figure 3D and Supplemental Figure 3A). The heart allograft also revealed a lower density of collagen I and fibronectin fibers in the CTLA-4–Ig + ACTH treatment group, as compared with the CTLA-4–Ig group (Figure 3E). Verhoeff’s staining of the heart allografts revealed thinner intima in the blood vessels of the CTLA-4–Ig + ACTH treatment group than the CTLA-4–Ig group (Figure 3F). These data indicate that the combination of CTLA-4–Ig + ACTH can reduce chronic fibrosis, a hallmark of chronic allograft rejection. Importantly, we also observed significant protection against CAV in our chronic rejection model.

We also found significantly higher gene expression of *Foxp3* in the heart allografts of the CTLA-4–Ig + ACTH treatment group versus the CTLA-4–Ig group by qPCR (Supplemental Figure 3B). The gene expression of the regulatory cytokine *Il10* trended higher in the CTLA-4–Ig + ACTH treatment group, while the gene expression of proinflammatory cytokines *Ifng*, *Tnfa*, and *Il17* trended higher in the CTLA-4–Ig group, and the chemokine receptor *Ccr2* was significantly higher in the CTLA-4–Ig group (Supplemental Figure 3B). CCR2 is a major proinflammatory chemokine receptor responsible for the trafficking of monocytic cells (56, 57). We also performed flow cytometry to evaluate the peripheral alloimmune response by collecting spleens and LNs from the transplant recipients at day 28. No significant difference in the Treg populations of the spleens and LNs was found between the 2 groups (Supplemental Figure 3, C and D). However, the Treg/Teff ratio was significantly higher in the spleens of the CTLA-4–Ig + ACTH group, as compared with the CTLA-4–Ig group (Supplemental Figure 3D).

### ACTH prolongs heart allograft survival in the absence of PI3Kγ.

**Figure 4:**
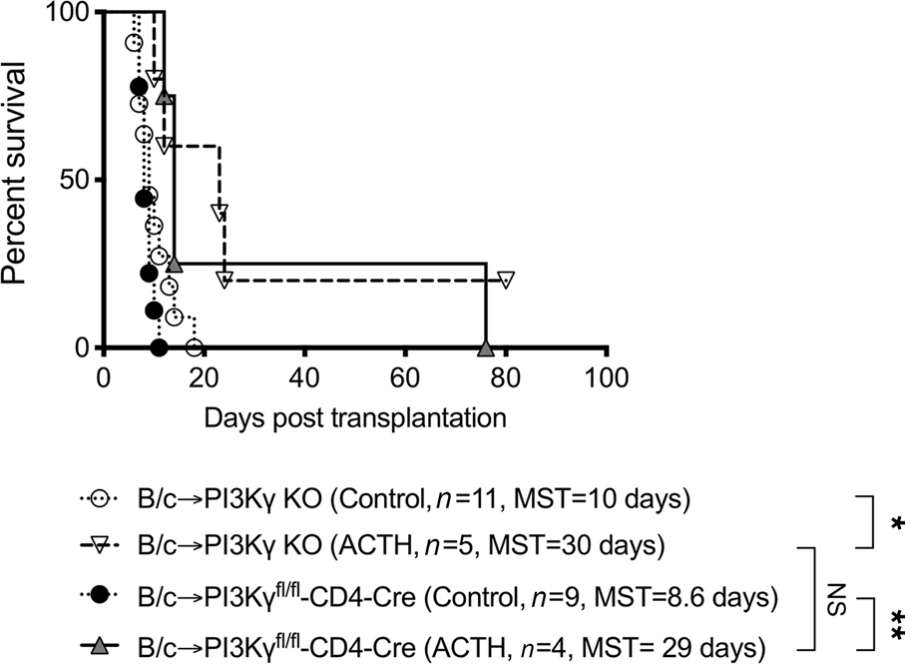
ACTH prolongs heart allograft survival in the absence of PI3Kγ. Comparison of heart allograft survival among *PI3K*γKO recipients of BALB/c hearts that received either ACTH (*n =* 5; MST, 30 days) or no treatment (Control; *n =* 11; MST, 10 days), and PI3Kγ^fl/fl^-CD4-Cre recipients of BALB/c hearts that received either ACTH (*n =* 4; MST, 29 days) or no treatment (control; *n =* 9; MST, 8.6 days). *P < 0.05, **P < 0.01 by log-rank test.

PI3Ks belong to a group of enzymes that phosphorylate lipids called phosphatidylinositols and regulate immune responses via activation of AKT (58, 59). We found in a previous study that low-dose CTLA-4–Ig synergized with PI3KγKO, resulting in indefinite prolongation of cardiac allograft survival (59). We sought here to investigate whether similar synergism occurs with ACTH treatment in the absence of PI3Kγ. As seen in Figure 4, ACTH treatment significantly prolonged heart allograft survival in PI3KγKO recipients, as compared with no treatment (MST, 30 days versus 10 days).

Inflammation has emerged as a key initiator of alloimmunity (60–63). As PI3Kγ is expressed by a wide range of immune cells (64–69), we next generated transgenic mice containing conditional KO of PI3Kγ in CD4^+^ T cells to pinpoint its importance in these cells. As shown in Figure 4, ACTH prolonged heart allograft survival significantly in PI3Kγ^fl/fl^-CD4-Cre recipients, as compared with no treatment (MST, 29 days versus 8.6 days). However, no significant difference in allograft survival between PI3Kγ^fl/fl^-CD4-Cre and PI3KγKO recipients that received ACTH was observed (MST, 29 days versus 30 days; Figure 4). These data indicate that the tolerogenic effects of ACTH occur mainly through regulation of CD4^+^ T cell function.

### ACTH ameliorates CAV.

**Figure 5:**
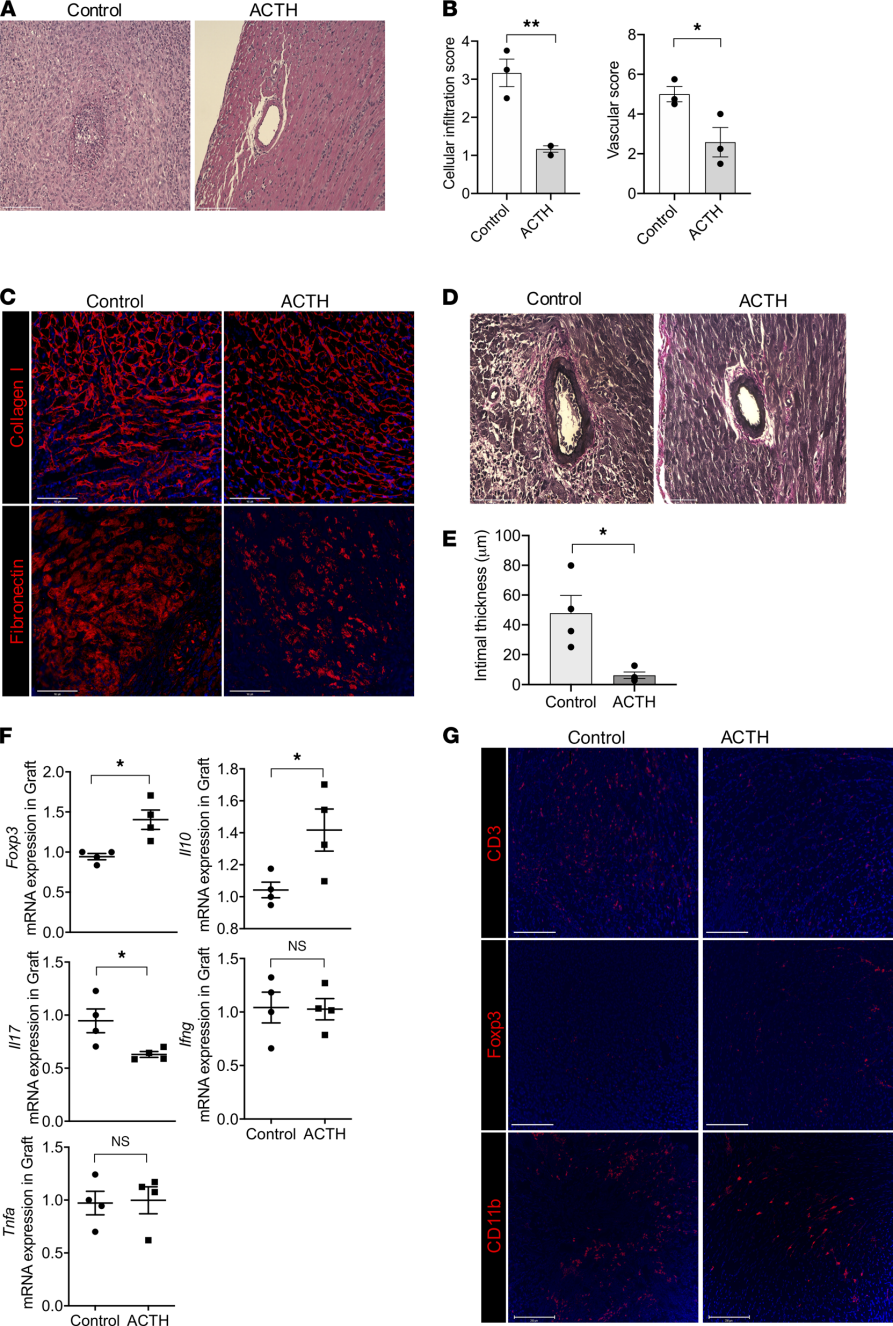
ACTH treatment ameliorated cardiac transplant vasculopathy. (**A**) Representative light micrographs of H&E-stained BM12 heart allograft sections around 4 weeks from C57BL/6 recipients following either ACTH treatment or no treatment (Control). Scale bar: 75 μm. (**B**) Comparison of cellular infiltration and vascular damage of the BM12 heart allografts from C57BL/6 recipients following either ACTH treatment or no treatment (Control) (*n =* 4 mice/group). (**C**) Representative fluorescence micrographs of fibronectin and collagen I fibers in the BM12 heart allografts from C57BL/6 recipients following either ACTH treatment or no treatment. Scale bar: 100 μm. (**D**) Light micrographs of Verhoeff’s-stained heart allograft sections from C57BL/6 recipients of BM12 heart allografts following either ACTH treatment or no treatment. Scale bar: 75 μm. (**E**) Bar graph shows comparison of intimal thickness in Verhoeff’s-stained BM12 heart allograft sections from C57BL/6 recipients following either ACTH treatment or no treatment (*n =* 4 mice/group). (**F**) Comparison of *Foxp3*, *Il10*, *Il17*, *Ifng*, and *Tnfa* gene expression between BM12 heart allografts in C57BL/6 recipients following either ACTH treatment or no treatment (Control) (*n =* 4 mice/group). (**G**) Representative fluorescence micrographs of CD3^+^ T cell, CD11b^+^ cell, and Foxp3^+^ Treg populations in BM12 heart allografts in C57BL/6 recipients following either ACTH treatment or no treatment (Control). Scale bar: 100 μm. Data presented as mean ± SEM; *P < 0.05, ***P < 0.001 by Student’s *t* test.

CAV is the primary cause of chronic heart transplant rejection. To assess the effect of ACTH on CAV, we used an MHC class II–mismatch model by transplanting hearts from BM12 mice into C57BL/6 recipients (BM12→C57BL/6). Heart allografts using this donor-recipient pairing exhibit long-term survival with no immunosuppression, as reported previously (70, 71). The mice were divided into an untreated control and an ACTH-treated group, and their heart allografts were collected around 4 weeks after transplantation. H&E staining of heart allografts revealed denser cellular infiltrates in the control group, whereas the ACTH-treated group contained significantly lower cellular infiltration and vascular injury, as indicated by semiquantitative assessment (Figure 5, A and B). As interstitial fibrosis is an important feature of chronic rejection, we stained the heart allograft for fibronectin and collagen I, and both markers revealed less fibrosis in the ACTH-treated group in comparison with the control group (Figure 5C). Verhoeff’s staining of the heart allografts revealed thinner intima in the blood vessels of the ACTH-treated group than the control group (Figure 5, D and E).

The gene expression levels of *Foxp3* and *Il10*, a major inducer of Tregs, were significantly higher in the heart allografts of the ACTH-treated mice (Figure 5F). Correspondingly, the expression of IL-17 was lower in the heart allografts of the ACTH-treated group in comparison with the control group (Figure 5F). IL-17 is a cytokine associated with inflammation, angiogenesis, and fibrosis, which are characteristic features of chronic heart transplant rejection (72–76). No significant difference in the gene expression of other proinflammatory cytokines, *Ifng* and *Tnfa*, was found (Figure 5F). Immunofluorescence staining revealed lower infiltration of CD3^+^ T cells and higher density of Foxp3^+^ Tregs in the heart allografts of the ACTH-treated group in comparison with the control group (Figure 5G). Spleens and LNs were collected from the transplant recipients for flow cytometric analysis of the peripheral immune response, and no difference in the percentages of Tregs or CD4^+^ IL-17^+^ T cells in both the spleens and LNs was found between the groups (Supplemental Figure 4, A and B). These data indicate that the graft itself is the main site of regulation of the alloimmune response (77–79). One possible side effect of ACTH treatment is hypercortisolism through unregulated stimulation of cortisol secretion by the adrenal gland. We measured serum cortisol levels in the mice to rule out this effect, and no significant difference was found between the untreated and ACTH-treated groups (data not shown).

## Discussion

Heart transplantation represents a lifesaving strategy for patients suffering from end-stage heart failure, but long-term success of the transplant is plagued by the development of chronic rejection (2–4, 80–82). Moreover, the conventional immunosuppressive agents used currently cause significant toxicity to several organ systems, increasing the risk of metabolic syndrome, diabetes, and kidney disease. Therefore, an exigent clinical need exists for the development of safer immunosuppressive agents aimed to lower the risk of chronic rejection in heart transplant recipients.

The importance of MCRs to diseases affecting diverse organ systems has been described and characterized (25, 83–86). Here, we used ACTH to interrogate the role of MCRs in the immune response, as we strove to identify an alternative signaling pathway for the delivery of immunosuppression following heart transplantation. Lymphocytes have been reported to express MC1R, MC3R, and MC5R (26, 27, 49, 87–90). However, the expression of MC2R by lymphocytes has not been explored. We demonstrated that human CD4^+^CD25^+^ T cells express MC2R more highly in comparison both to other lymphocyte subsets as well as to other MCRs. Moreover, the gene expression of MC2R was significantly higher in human Tregs than in naive T cells. In addition, ACTH enhanced the production of MRAP2, a protein necessary to the proper functioning of MC2R. The use of an MC2R blocker blunted the differentiation of human PBMCs to Tregs in a Treg induction assay, thereby confirming the importance of MC2R to the immunosuppressive effects of ACTH on human cells. We observed similar features in lymphocytes from WT and CD28KO mice, as ACTH increased the synthesis of Tregs significantly in vitro.

IL-2 is crucial for the maintenance of the Treg lineage, as well as their proper immunosuppressive function (91). Although IL-2 is not required for the survival of Tregs, its absence is associated with marked impairment of their function, as well as with the development of autoimmunity (92). The receptor for IL-2 is composed of 3 subunits: IL-2Rα (CD25), IL-2Rβ (CD122), and common c-chain (cc) (CD132). Of these, IL-2Rα is expressed constitutively by Tregs. Shatrova et al. have identified the JAK3/STAT5 signaling pathway as a major molecular cascade for signal transduction following interaction of IL-2 with IL-2Rα on the membrane of human peripheral blood lymphocytes, and activation of this pathway resulted in increased expression of IL-2Rα in a feed-forward mechanism (93). Other studies have also highlighted the importance of STAT5 activity downstream of IL-2R signaling in upregulating the expression of IL-2Rα in T cells (94) and Tregs (95), as STAT5 interacts with Foxp3 and the transcription factor Runx1 in the latter population (96, 97). However, the relationship between MC2R signaling and IL-2Rα expression is unknown. Here, we found that IL-2Rα is expressed more highly following exposure of lymphocytes to ACTH in a Treg induction assay, unveiling a possible consequence of MC2R activation. We also found that ACTH increases the expression of p-STAT5, a mediator of responsiveness to IL-2 (98). Together, these findings suggest that ACTH increases the expression of IL-2Rα by Tregs through binding to MC2R, thereby potentially increasing their sensitivity toward IL-2. Though we found that the ACTH/MC2R pathway plays a pivotal role in alloimmunity, we will need to decipher the relative roles of other MCRs that respond to ACTH, through the use of other conditional KO mice in the future. We also plan to interrogate the importance of the JAK3/STAT5 signaling pathway to the propagation of ACTH-induced changes in Tregs, through the use of specific inhibitors. We are also very much interested in understanding the effect of IL-2 on the proliferation of ACTH-treated Tregs, and we will pursue the question of this impact in future studies. We also plan to examine the dependence of ACTH-mediated survival prolongation on IL-2 and to interrogate the source of IL-2 in future experiments.

The CD28 costimulatory pathway has been studied for many years in transplantation (99). In fact, CD28KO mice have been used widely to elucidate the CD28/B7 signaling pathway, and CTLA-4-Ig — an antagonist of the CD28 signaling pathway — has been used to prevent allograft rejection in transplantation. However, a significant concern related to the mechanism of action for CTLA-4–Ig is that it can inhibit the activation of Tregs alongside Teffs, which may limit its efficacy in immunosuppression. Therefore, further investigation is required to assess the stability, in vivo function, and survival of Tregs generated under CD28 blockade.

After establishing that ACTH treatment induces the production of Tregs in vitro, we decided to examine whether this antiinflammatory effect applied to a mouse model of allogeneic heart transplantation. Here, we report for the first time to our knowledge that ACTH prolongs heart allograft survival in CD28KO mouse recipients, but it has no effect on WT recipients. Moreover, ACTH treatment increased the ratio between Tregs and Teffs in the spleens of the CD28KO mice, substantiating the concept that ACTH promotes the differentiation of Tregs through MC2R, a signaling pathway that likely does not require activity of CD28. Due to the possibility of its antagonism toward Tregs, we decided to limit the administration of CTLA-4–Ig to a low dose in our study. Our results confirmed that the combination of ACTH with low-dose CTLA-4–Ig supported tolerance of the heart allograft, a process mediated significantly by support of Tregs by ACTH.

Our results also show that PI3Kγ inhibition could constitute another strategy to favor the activity of Tregs over Teffs. Due to its preferential enrichment in leukocytes, the γ catalytic form of PI3K has gained attention for its role in regulating the function of immune cells and as a promising drug target for the treatment of inflammatory diseases (64, 100–108). We have shown previously that PI3Kγ inhibition synergizes with CTLA-4-Ig, representing a promising strategy to increase heart allograft acceptance (59). The PI3Kγ Inhibitor IPI-549 is administered to patients currently in several clinical trials (NCT03980041, NCT02637531, NCT03961698, NCT03795610). In future experiments, we will add this inhibitor to combination treatment with ACTH and CTLA-4–Ig. We will also test whether combination treatment with ACTH and IL-2 promotes Tregs and prolongs cardiac allograft survival.

CAV is the primary cause of heart transplant failure, and interstitial fibrosis is a major consequence of chronic allograft injury (80, 82). We found that interstitial fibrosis of heart allografts in WT mice was lower following treatment with the combination of ACTH with low-dose CTLA-4-Ig, as compared with ACTH alone. From a clinical standpoint, improvement of long-term transplant outcomes through the introduction of safer combinatorial immune therapies that enable the targeting of specific effector alloimmune responses is a major unmet need. Such therapies ideally should not cause activation of proinflammatory innate immune responses, but they should also promote the regulatory axis. To fulfill these unmet clinical needs, we have demonstrated that ACTH synergizes with CTLA-4–Ig and the effects of PI3Kγ and CD28 deficiency. Collectively, these data support a potentially new immunomodulatory strategy for cardiac transplant that includes ACTH, CTLA-4–Ig inhibition, and PI3Kγ inhibition. Intriguingly, PI3Kγ inhibition also has antineoplastic effects, which makes it highly suitable for transplant recipients who are at elevated risk for cancer, due to chronic immunosuppression (109, 110).

The major physiological role of ACTH is to induce the production of glucocorticoids by the adrenal cortex. Although we demonstrated in this study that serum cortisol levels in the ACTH-treated mice were not elevated, the blood was drawn from these mice several weeks following the last dose of ACTH, so its effect on secretion of cortisol may have diminished at this point. ACTH has been shown previously not only to decrease clinical disease, suppress IL-17 production, and inhibit encephalitogenic Th1-like secretion of IFN-γ, but also to increase Treg frequency in an experimental autoimmune encephalitis mouse model (111). On the other hand, the importance of glucocorticoids in direct promotion of Tregs remains a matter of debate. Administration of systemic and inhaled glucocorticoids to human asthma patients was correlated with an increase in circulating Tregs (112). This positive correlation between glucocorticoid use and circulating Treg population was replicated in several other small human studies of autoimmune diseases, including systemic lupus erythematosus, multiple sclerosis, myasthenia gravis, and idiopathic thrombocytopenic purpura (113–116). However, 2 larger, more comprehensive human studies of patients with asthma and connective tissue diseases revealed the opposite observation that glucocorticoid use actually decreased the population of circulating Tregs (117, 118). Therefore, though we have demonstrated that ACTH directly induces the differentiation of T cells into Tregs in this study, potential confounding of the in vivo findings by glucocorticoid activity is possible.

One possible method to reduce the effect of steroids is to administer a selective glucocorticoid receptor antagonist, such as RU-43044, simultaneously with ACTH to the recipient mice following heart transplantation (119). Then, the effects of glucocorticoid antagonism on allograft survival, cellular infiltration of the allograft, Treg/Teff ratio, and pro- as well as antiinflammatory cytokine production in the serum can be measured. Another possible method to diminish the effect of steroids due to MC2R activation in the adrenal cortex would be to employ an adrenal cortex–specific conditional KO mouse strain. Please note that complete KOs of MC2R and MRAP are lethal at birth (120), and no standard transgenic animal model of adrenal insufficiency currently exists, to our knowledge. In future studies, we could generate a conditional KO of melanocortin-2 receptor accessory protein (MRAP) in SF1-Cre mice, for which scientists have ablated gene expression in the adrenal cortex successfully in the past (121). However, the likelihood that this KO will be lethal is high.

Previous studies have demonstrated that CD4^+^IL-17^+^ cells constitute a subset of donor-reactive T cells and make critical contributions to allograft rejection (122, 123). Similarly, alloreactive Tregs are crucial mediators of the tolerogenic response following transplantation (124, 125). On the basis of these prior findings, we used the Th17 cell and Treg populations in the allografts as surrogates to define the balance between the allogenic and tolerogenic arms of the immune response among alloreactive T cells in the transplant recipient. However, future studies in which Th17 cells and Tregs are depleted are required to identify more precisely the direct roles of Tregs and Th17 cells in donor reactivity.

**Figure 6:**
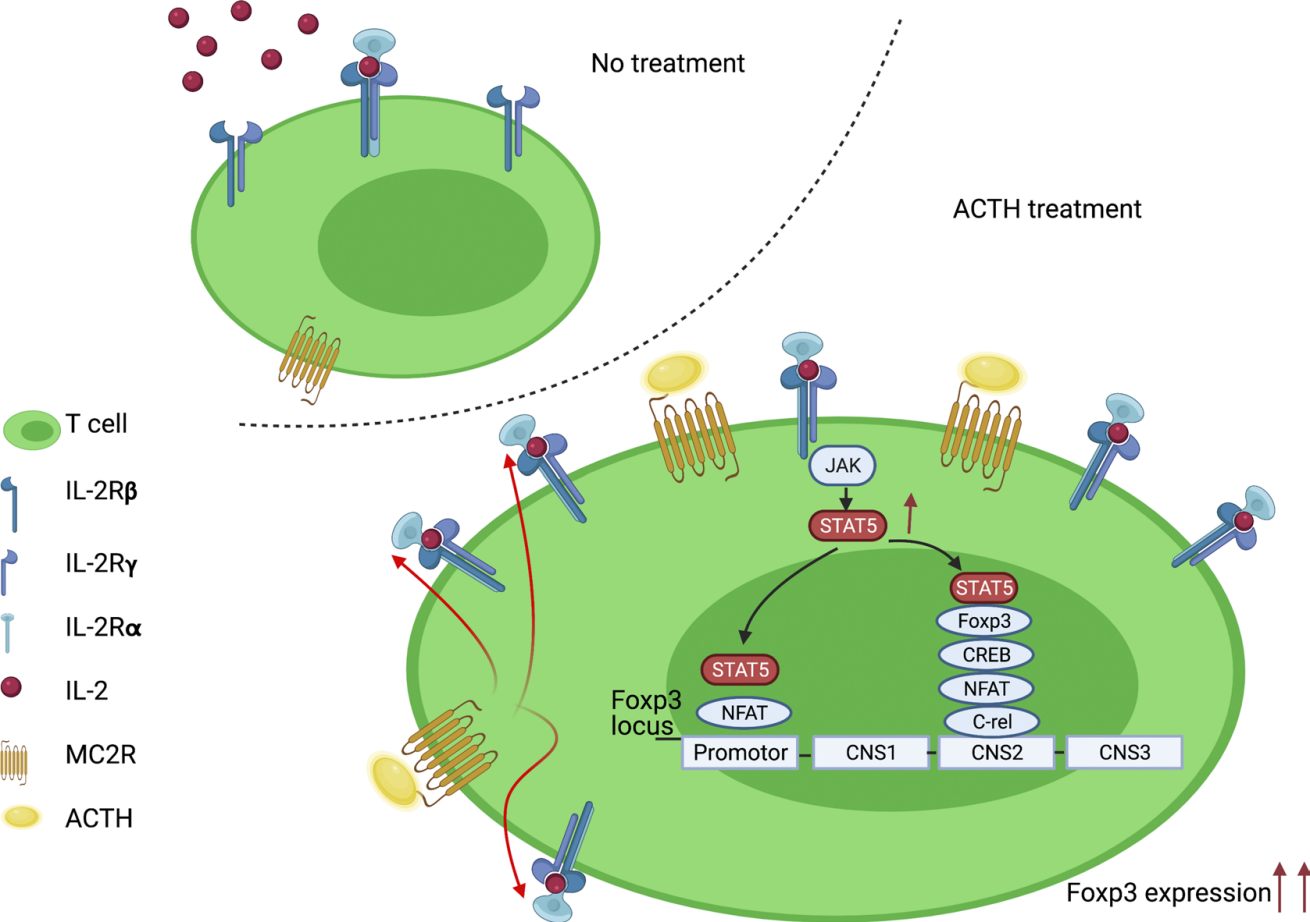
Schematic of effect of ACTH treatment in T cells. Binding of ACTH to MC2R stimulates expression of IL-2Rα and activation of its intracellular signaling pathway, resulting in Foxp3 expression and differentiation of CD4^+^ T cells into Tregs (Created with BioRender.com).

In summary, our investigation suggests that ACTH can boost heart allograft tolerance by activating Tregs through its ligand MC2R. This effect is mediated at least partially by increasing the expression by Tregs of IL-2Rα, possibly increasing their sensitivity to IL-2 and the activity of STAT5, which is a major inducer of Foxp3 (Figure 6). Clinically, ACTH synergizes with inhibition of CTLA-4–Ig and absence of PI3Kγ activity to augment immunosuppression and reduce chronic rejection markedly. Therefore, these findings provide an avenue for future testing of ACTH and its analogs as adjunct immunosuppressive agents to reduce the incidence of rejection following heart transplantation.

## Methods

### Mice.

Seven- to 8-week-old WT C57BL/6J (C57BL/6 or WT, The Jackson Laboratory, stock no. 00064), B6(c)-H2-Ab1^bm12^/KhEgJ (BM12, The Jackson Laboratory, stock no. 001162), B6.129S2-*Cd28^tm1Mak^*/J (CD28KO, The Jackson Laboratory, stock no. 002666), BALB/cByJ (BALB/c, The Jackson Laboratory, stock no. 001026), B6.129S7-*Rag1^tm1Mom^*/J (RAGKO, The Jackson Laboratory, stock no. 002216), B6N.Cg-Tg(ACTFLPe)9205Dym/CjDswJ (FLPe B6N, The Jackson Laboratory, stock no. 019100), and B6.Cg-Tg(Cd4-Cre)1Cwi/BfluJ (CD4-Cre, The Jackson Laboratory, stock no. 022071) mice were used. *PI3K*γ*^−/−^* C57BL/6 mice (*PI3K*γ*KO*, backcrossed 11 generations) were received from Bao Lu (Boston Children’s Hospital/Harvard Medical School, Boston, Massachusetts, USA) and maintained in our animal facility (126).

Pik3cg^tm1a(EUCOMM)Wtsi^ (Pik3cg-tm1a) mice were purchased from Wellcome Trust Sanger Institute (Hinxton, United Kingdom). Male and female mice were housed in sterilized and ventilated cages in a specific pathogen–free animal facility under a standard 12-hour light/12-hour dark cycle. Mice were fed irradiated food and water ad libitum. All experiments were performed with age- and sex-matched, 8- to 12-week-old mice in accordance with the relevant guidelines and regulations approved by the IACUC of Brigham and Women’s Hospital.

### Creation of PI3Kγ^fl/fl^-CD4-Cre mice.

To obtain a conditional KO mouse, we first created PI3K*γ*^fl/fl^ mice by crossing Pik3cg-tm1a with FLPe B6N mice. The genotypes of these mice were checked by PCR, using the following primers: *5rft*_forward (*5rft*_F): 5′-AGGCGATAACGATACCACGAT-3′; *5rft*_reverse (*5rft*_R): 5′-CCACAACGGGTTCTTCTGTT-3′; *tm1c*_F: 5′-AAGGCGCATAACGATACCAC-3′; *tm1c*_R: 5′-CCGCCTACTGCGACTATAGAGA-3′; *Lacz_2_small*_F: 5′-ATCACGACGCGCTGTATC-3′; and *Lacz_2_small*_R: 5′-ACATCGGGCAAATAATATCG-3′. Then, we crossed PI3Kγ^fl/fl^ (Pik3cg-tm1c) mice with CD4-Cre mice to create PI3Kγ^fl/fl^-CD4-Cre (Pik3cg-tm1d-CD4-Cre) mice. The genotypes of these mice were checked by PCR, using the following primers: *Cre*_F: 5′-CATTTGGGCCAGCTAAACAT-3′; *Cre*_R: 5′-TAAGCAATCCCCAGAAATGC-3′; *Cas_r1*_Term: 5′-TCGTGGTATCGTTATGCGCC-3′; *Floxed_*PNF: 5′-ATCCGGGGGTACCGCGTCGAG-3′; *Floxed_*LR: 5′-ACTGATGGCGAGCTCAGACC-3′; *Tm1b_prom*_F: 5′-CGGTCGCTACCATTACCAGT-3′; *Tm1c*_F: 5′-AAGGCGCATAACGATACCAC-3′.

### Mouse heterotopic cardiac transplantation.

Vascularized intraabdominal heterotopic transplantation of heart allografts was performed using microsurgical techniques, as previously described (60, 61, 81). Briefly, the ascending aorta and pulmonary artery of the donor mouse were sutured to the abdominal aorta and inferior vena cava of the recipient mouse separately by using 10-0 sutures. The status of the heart allograft was monitored daily by abdominal palpation. Rejection was defined as complete cessation of a palpable heartbeat and confirmed by direct visualization at laparotomy. Loss of graft function within 24 hours of transplant was considered a technical failure (<10%, on average), and these mice were omitted from further analysis.

### In vivo treatment protocol.

Rat ACTH (1–39) was purchased from GenScript. ACTH was administered daily in 18 μg doses by i.v. injection on day 0 through day 7 after transplantation. Abatacept (CTLA-4–Ig) (Orencia) was purchased from Bristol-Myers Squibb; the mice received a single 250 μg i.p. dose on day 2 after transplantation.

### T cell isolation from mice and Treg induction.

CD4^+^CD25^–^ cells were isolated from the spleens of mice by MACS using EASYSEP Magnets (Stemcell Technologies), according to the manufacturer’s protocol and placed in a 96-well plate. Tregs were induced by addition of anti–mouse CD3 (clone 145-2C11, eBioscience) and CD28 (clone 37.51, eBioscience) antibodies (3 μg/mL in complete RPMI media), TGF-β (10 ng/mL, BioLegend) and IL-2 (100 ng/mL, BioLegend) cytokines, and ACTH (100ng/mL, MilliporeSigma). The plate was incubated in 5% CO_2_ at 37°C for 3 days.

### Isolation of human peripheral blood mononuclear cells (PBMCs).

Unidentified apheresis blood samples were obtained from healthy human donors and processed within 24 hours of blood extraction. Blood samples from healthy volunteers were obtained after informed, signed consent. PBMCs were isolated by density gradient centrifugation at 1200*g* for 15 minutes at 4°C, using Lymphoprep density gradient medium and SepMate tubes (Stemcell Technologies).

### Human CD4^+^CD25^–^ responder T cell isolation and culture.

CD4^+^CD25^–^ T cells, isolated by MACS from freshly separated PBMCs using EASYSEP Magnets (Stemcell Technologies) by the manufacturer’s protocol, were cultured in complete medium (Corning RPMI 1640) with 10% pooled human AB serum (GemCell U.S. Origin Human Serum AB), 1% L-glutamine, and 1% penicillin-streptomycin. Functional-grade purified anti–human CD3 (OKT3) and functional-grade purified anti–human CD28 (CD28.2) (both from eBioscience) were added at 2 μg/mL. Recombinant IL-2 (200 ng/mL, Peprotech), TGF-β (5 ng/mL), ACTH (100-1000 ng/mL, MilliporeSigma), and MC2R blocker (400 nM/mL, MyBioSource) were also added.

### Flow cytometry.

Antibodies were purchased from BioLegend, unless otherwise stated. Human antibodies included CD4 (RPA-T4), CD25 (M-A251), and Foxp3 (236A/E7) (Thermo Fisher Scientific). Mouse antibodies included CD4 (RM4-5), CD8a (53-6.7), CD25 (PC61), CD44 (IM7), CD62L (MEL-14), Foxp3 (MF-14), and CD11b (M1/70). For intracellular cytokine staining, cells were stimulated with phorbol 12-myristate 13-acetase (PMA, 50 ng/mL) and ionomycin (500 ng/mL) in combination with GolgiStop for 4 hours; they were then permeabilized and stained with anti–IFN-γ (XMG1.2) and anti–IL-17A (TC11-18H10.1) antibodies. Fluorescence was detected by a BD FACSCanto II (BD Biosciences) flow cytometer. Data was analyzed using FlowJo software.

### ELIspot.

ELIspot assay was performed according to the manufacturer’s instructions (BD Biosciences) to measure the production of IFN-γ. Briefly, an immunospot plate (MilliporeSigma) was coated with IFN-γ primary antibody for 3 hours in 37°C. Donor (BALB/c) splenocytes were irradiated at 3000 Rads, plated with recipient (CD28KO) splenocytes in a 1:1 ratio, and incubated at 37°C for 24 hours. Cells were then washed and incubated overnight with the secondary antibody. After development with the chromogen, the total number of spots per well was quantified using an ImmunoSpot Analyzer.

### MLR assay.

Irradiated donor (BALB/c) splenocytes (stimulator) and recipient (CD28KO) splenocytes (responder) were added to each well of a 96-well round-bottom plate and incubated at 37°C for 2 days. In total, 1 μCi of tritiated thymidine (^3^H) was added, and the plate was incubated for additional 14 hours. The cells were transferred to a filter map by a Tomtec Harvester 96-cell harvester and analyzed by a 1450 MicroBeta TriLux microplate scintillation counter.

### Luminex assay for quantification of cytokines.

Cytokines were measured in the supernatant using the MILLIPLEX MAP Mouse Cytokine/Chemokine Magnetic Kit (MilliporeSigma), as per the manufacturer’s instructions. Mean fluorescence intensity (MFI) was measured by a Luminex 200 IS instrument and analyzed using the logistic curve-fitting method to determine cytokine concentrations.

### Serum cortisol measurement.

Serum from mice were collected. Cortisol levels were measured using the Elecsys immunoassay system on the Cobas E601 immunology analyzer (both from Roche Diagnostics).

### Quantitative PCR (qPCR).

RNA was isolated with TRIZOL (Invitrogen), and cDNA was synthesized using 2 μg of RNA and High-Capacity Reverse Transcriptase (Invitrogen), as described previously (60). Reverse transcription PCR (RT-PCR) was performed with SYBR Green PCR reagents on a Bio-Rad detection system. RNA levels were normalized to the level of GAPDH and calculated as ΔΔCT. Primers used for RT-PCR are listed as below: *Gapdh*_F: 5′-AGCCACATCGCTCAGACAC-3′, *Gapdh*_R: 5′-GCCCAATACGACCAAATCC-3′; *Foxp3*_F: 5′-GGCCCTTCTCCAGGACAGA-3′, *Foxp3*_R: 5′-GCTGATCATGGCTGGGTTGT-3′; *Il10*_F: 5′-GCTCTTACTGACTGGCATGAG-3′, *Il10*_R: 5′-CGCAGCTCTAGGAGCATGTG-3′; *Ifng*_F: 5′-TTGAGGTCAACAACCCACAG-3′, *Ifng*_R: 5′-TCAGCAGCGACTCCTTTTC-3′; *Il2*_F: 5′-TGAGCAGGATGGAGAATTACAG-3′, *Il2*_R: 5′-GTCCAAGTTCATCTTCTAGGCAC-3′; *Tnfa*_F: 5′-ATGAGAAGTTCCCAAATGGC-3′, *Tnfa*_R: 5′-CTCCACTTGGTGGTTTGCTA-3′; *Il2ra*_F: 5′-CCACATTCAAAGCCCTCTCCTA-3′, *Il2ra*_R: 5′-AGTTGCTGGTGCACTGGCAG-3′; *Il17*_F: 5′-AAGGCAGCAGCGATCATCC-3′, *Il17*_R: 5′-GGAACGGTTGAGGTAGTCTGAG-3′; *Ccr2*_F: 5′-ACACCCTGTTTCGCTGTAGG-3′, *Ccr2*_R: 5′-GATTCCTGGAAGGTGGTCAA-3′; *Nfatc2*_F: 5′-GCAGTCCCCAAGACGAGCTG, *Nfatc2*_R: 5′-CTATACTATCCGGCTCTCCGAACCG-3′; *Ap1*_F: 5′-CATCTACAGTGGGGCCGA-3′, *Ap1*_R: 5′-ATGCCTTAGCTGGGGTCA-3′; *E2a*_F: 5′-GGCTGGAGATGTTGAGAGCAA-3′, *E2a*_R: 5′-AAAGGAAATCCAGTGCGC-3′; *Crel*_F: 5′-CGAACCCAATTTATGACAACCG-3′, *Crel*_R: 5′-TTTTGTTTCTTTGCTTTATTGCCG-3′; *Il1b*_F: 5′-CTGTGTCTTTCCCGTGGACC-3′, *Il1b*_R: 5′-CAGCTCATATGGGTCCGACA-3′; hu-GAPDH_F: 5′-GGATTTGGTCGTATTGGG-3′, hu-GAPDH_R: 5′-GGAAGATGGTGATGGGA-3′; hu-MC1R_F: 5′-GAGGGAGCTGAGGACCAGGC-3′, hu-MC1R_R: 5′-TTCCATCTGGGCACCCCCAG-3′; hu-MC2R_F: 5′-GAGCTCAGGGACGCATTCAA-3′, hu-MC2R_R: 5′-CCTTGCATCCATTAGGGAAG-3′; hu-MC3R_F: 5′-ACCTTCGAGGACCAGTTTAT-3′, hu-MC3R_R: 5′-GTGAGGGCCTTCCTCACGGT-3′; hu-MC4R_F: 5′-GTGAATATTGATAATGTCAT-3′, hu-MC4R_R: 5′-AACTTATGATGATCCCAACC-3′; hu-MC5R_F: 5′-GTGTTTACCGTGTGCTGGGC-3′, hu-MC5R_R: 5′-ATATATGAGAGGGTCCATCA-3′. All RT-PCR reactions were performed in triplicate.

### Western blot and quantification.

To perform the Western blot analysis for Tregs and naive T cells, cells were harvested and washed with PBS, and they were lysed with RIPA buffer supplemented with freshly added protease inhibitor cocktail (4693159001, Roche). The lysates were run on Criterion SDS-PAGE gels (Bio-Rad), transferred to PVDF membranes, and analyzed by Western blot, using the following rabbit antibodies: p-STAT5 (9351s, Cell Signaling Technology) and MRAP2 (MRAP2-201AP, FabGennix International). Membranes were incubated with horseradish peroxidase–labeled secondary antibodies, and bands were visualized with enhanced chemiluminescence (Advansta). Quantification of bands was performed using ImageJ (NCBI, 1.8.0_112), normalized to GAPDH, and assessed in triplicates.

### IHC, immunofluorescence staining, and quantification.

Cells were stained with conjugated or purified antibodies. Purified antibodies were detected using secondary antibodies. The antibodies included anti-Foxp3 (126401, BioLegend), anti-MC1R (LS-C332324-50, LSBio), anti-MC2R (LS-C164069, LSBio), anti-MC3R (MAB3737-SP, R&D Systems), and anti-MC5R (ab133656, Abcam). DAPI (VECTASHIELD, Vector Laboratories) was used to counterstain the cell nuclei. The stained cells were visualized by an EVOSTM FL Auto 2 Imaging System (Thermo Fisher Scientific).

Heart allografts were harvested at designated time points after transplant. Heart allografts were fixed in formalin, embedded in a paraffin block or preserved in Optimal Cutting Temperature (OCT) compound (Tissue-Tek), and stored at –80°C. Samples were cut into 5 μm sections and stained with H&E and Verhoeff’s stain (paraffin block section). For IF staining, sections were stained with conjugated or purified antibodies. Purified antibodies were detected using secondary antibodies. The antibodies included anti-CD11b (101202, BioLegend), anti–collagen I (ab34710, Abcam), anti-fibronectin (ab2413, Abcam), anti-Foxp3 (126401, BioLegend), and anti-CD3 (100201, BioLegend). DAPI (VECTASHIELD, Vector Laboratories) was used to counterstain the cell nuclei. The stained tissue sections were visualized using an EVOS FL Auto 2 Imaging System (Thermo Fisher Scientific). Quantification was performed on 4–5 sections from at least 3 separate mice using Celleste (Invitrogen) and ImageJ (NCBI, 1.8.0_112) image analysis software.

### Histological scoring and measurement.

Histological scoring of H&E-stained heart allografts was performed by a modified method from the International Society for Heart and Lung Transplantation (127, 128), as described previously (60). Briefly, all scoring was performed blindly on 6 random microscopic fields for each heart section (6 sections/heart, 3–4 mice per group). Cellular infiltration was graded from 0 to 4. Vascular appearance was determined by a combination of vascular occlusion score and perivascular cellular infiltration. Vascular (artery) occlusion was scored from grade 0 to 3 for every artery. The sum of the vascular occlusion score and perivascular cellular infiltration score was designated as the vascular appearance score. Intimal thickness of heart allografts was measured by Verhoeff’s stain.

### Statistics.

All data were analyzed by GraphPad Prism 8 (GraphPad Prism Software Inc.) and presented as the mean ± SEM. Two-tailed Student’s *t* test, Mann-Whitney *U* test, or 1-way ANOVA was performed to determine significant differences among groups. A statistical evaluation of graft survival was analyzed by the Kaplan-Meier curve, and the log rank test was performed to determine the effect. Significance was defined as *P <* 0.05.

### Study approval.

All animal experiments were approved and performed in accordance with the guidelines and regulations of the IACUC of the Brigham and Women’s Hospital and Harvard Medical School. Healthy human donors were recruited after obtaining informed, signed consent. The study protocol was reviewed and approved by an IRB at the Brigham and Women’s Hospital and was conducted in full conformance with the principles of the Declaration of Helsinki.

## Author contributions

JZ designed and performed experiments, analyzed and interpreted data, and drafted the manuscript. JL performed the immunofluorescent staining, RT-PCR, and Western blot; analyzed data; and modified the manuscript. JZ and MU performed microsurgery. NB, BSAD, TI, and XL performed ELIspot, immunofluorescent staining, and flow cytometry and analyzed data. JA, PJ, PF, SGT, and JCM helped with the study design. VK modified the main text and critically revised the manuscript. RA designed the study, interpreted the data, and critically revised and finalized the manuscript. All authors edited and approved the manuscript. Order of co-first and co-senior authors was decided on the bases of the time and effort of their relative contributions made toward the project and manuscript. All authors approved of this order.

## Supplementary Material

Supplemental data

## Version 1 07/08/2021

Electronic publication
