# Supplementary material for: ACTH treatment promotes murine cardiac allograft acceptance
Source: JCI Insight. 2021 Jul 8;6(13):e143385. doi: 10.1172/jci.insight.143385 (PMC8410061; doi:10.1172/jci.insight.143385)
Supplement: Supplemental data [file jciinsight-6-143385-s052.pdf]

ACTH treatment promotes murine cardiac allograft acceptance

Jing Zhao[1,2†], Liwei Jiang[1,2†], Mayuko Uehara[1,2], Naima Banouni[1,2], Basmah S Al Dulaijan[1,2], Jamil Azzi[1,2], Takaharu Ichimura[2], Xiaofei Li[1,2], Petr Jarolim[3], Paolo Fiorina[4], Stefan G.Tullius[5], Joren C. Madsen[6,7], Vivek Kasinath[1,2‡], Reza Abdi[1,2‡]

[1]Transplantation Research Center, Brigham and Women's Hospital, Harvard Medical School, Boston, MA 02115, USA

[2]Renal Division, Brigham and Women's Hospital, Harvard Medical School, Boston, MA 02115, USA

[3]Department of Pathology, Brigham and Women's Hospital, Harvard Medical School, Boston, MA 02115, USA

[4]Department of Nephrology, Boston Children's Hospital, Harvard Medical School, Boston, MA 02115, USA

[5]Division of Transplant Surgery, Department of Surgery, Brigham and Women's Hospital, Harvard Medical School, Boston, MA, 02115, USA

[6]Center for Transplantation Sciences, Department of Surgery, Massachusetts General Hospital and Harvard Medical School, Boston, MA, 02114USA

[7]Division of Cardiac Surgery, Department of Surgery, Massachusetts General Hospital and Harvard Medical School, Boston, MA, 02114, USA

Correspondence:

Reza Abdi, MD

Transplantation Research Center, Brigham and Women's Hospital, 221 Longwood Ave, Boston MA 02115, USA

Tel: 617-732-5259, Fax: 617-732-5254, Email: rabdi@rics.bwh.harvard.edu

† Co-first authors; ‡ co-senior authors

Conflict-of-interest: The authors have declared that no conflict of interest exists.

Supplemental Figures and Figure Legends:

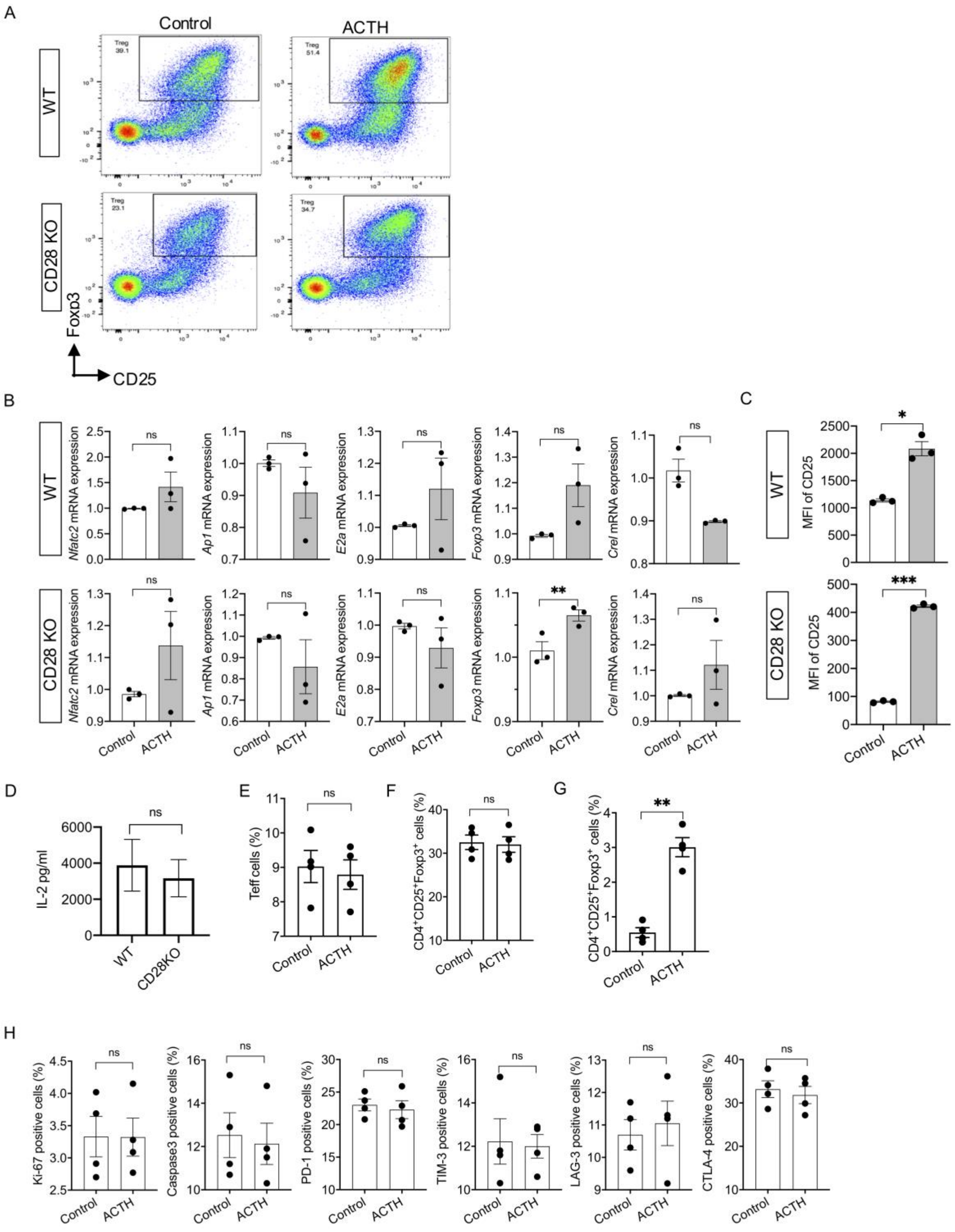

Supplemental Figure 1. (A) Representative flow cytometry plots of $CD4^+CD25^+Foxp3^+$ Treg

populations from Treg assay in untreated control and ACTH-treated groups, using lymphocytes from Wild-type (WT) and CD28 knock-out (CD28KO) C57BL/6 background mice. (B) qPCR of Tregs shows significantly increased *Foxp3* gene expression in ACTH-treated group compared to untreated control group from CD28KO mice, but no significant difference is seen with WT mice. No significant difference in gene expression levels of *Nfatc2*, *Ap1*, E2a, and *Cre1* between untreated control and ACTH-treated group from WT and CD28KO mice was seen. (C) Flow cytometric analysis reveals that ACTH induced a higher CD25 expression (by mean fluorescence intensity (MFI)) than no treatment (control) in a Treg induction assay, using splenocytes from WT and CD28KO mice. Experiments were performed independently in triplicate. (D) Luminex assay revealed no significant difference in IL-2 concentration between WT and CD28KO mice following ACTH treatment. (E) Flow cytometric analysis of T cells from WT spleen shows no difference of percentage in Teff cells following either treatment with ACTH or no treatment (Control). (F) Flow cytometric analysis of T cells collected from WT spleen shows no significant difference in the percentages of nature Tregs (nTregs) between the ACTH-treated group and the untreated control group. (G) Flow cytometric analysis of T cells collected from WT spleen shows significantly higher percentage of induced Tregs (iTregs) in ACTH-treated group than untreated control group. (H) Flow cytometric analysis of Tregs collected from WT spleen shows no significant difference in the percentages of Ki-67, Caspase 3, PD-1, TIM-3, LAG-3 and CTLA-4 between the ACTH-treated group and untreated control group. Data presented as the mean ± SEM, *p<0.05, ** p<0.01, *** p<0.001.

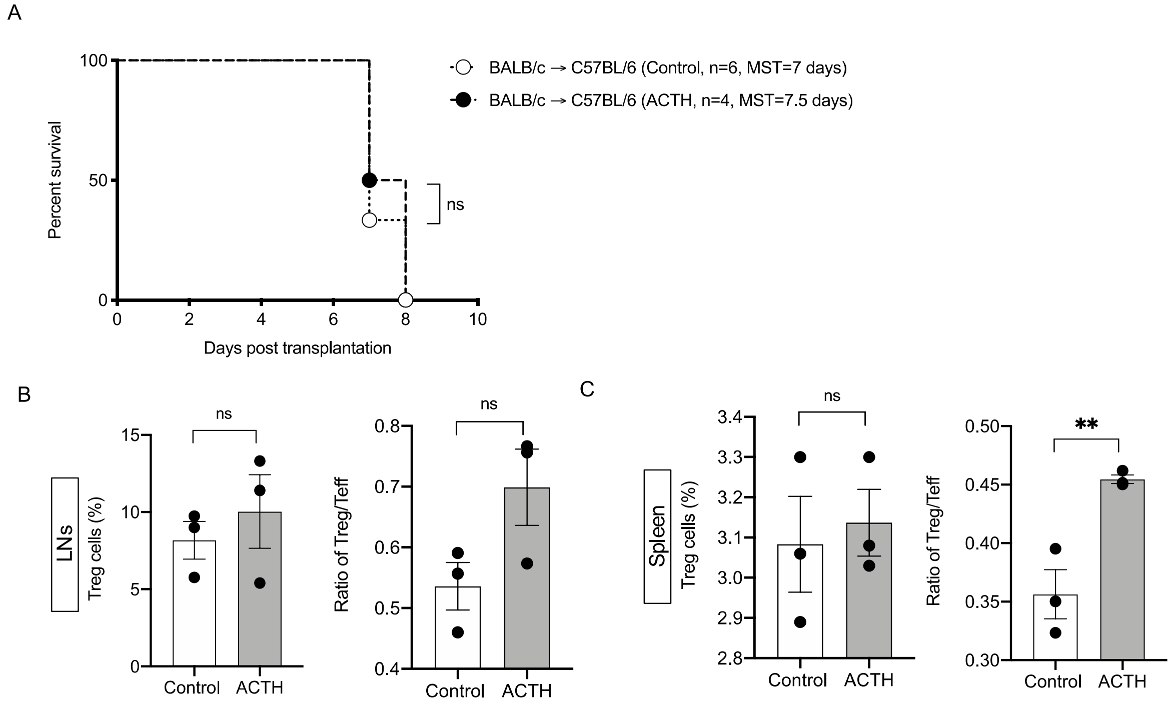

Supplemental Figure 2. (A) Survival curve demonstrates no significant difference in heart allograft survival between C57BL/6 recipients of BALB/c hearts that received ACTH (n=4 mice/group, MST=7.5 days) versus untreated allograft recipients (control; n= 6 mice/group, MST=7 days). (B) Flow cytometric analysis of LNs collected from CD28KO recipients at Day 7 shows no difference in percentages of Tregs and Treg/Teff ratio (CD4$^+$CD25$^+$Foxp3$^+$/ CD44$^+$CD62L$^-$) between the ACTH-treated group and untreated control group. (C) Flow cytometric analysis of spleens collected from CD28KO recipients at day 7 shows no significant difference in Tregs but significantly higher Treg/Teff ratio in the ACTH-treated group than the untreated control group. (n=3 mice/group) Data presented as the mean ± SEM, ** $p<0.01$.

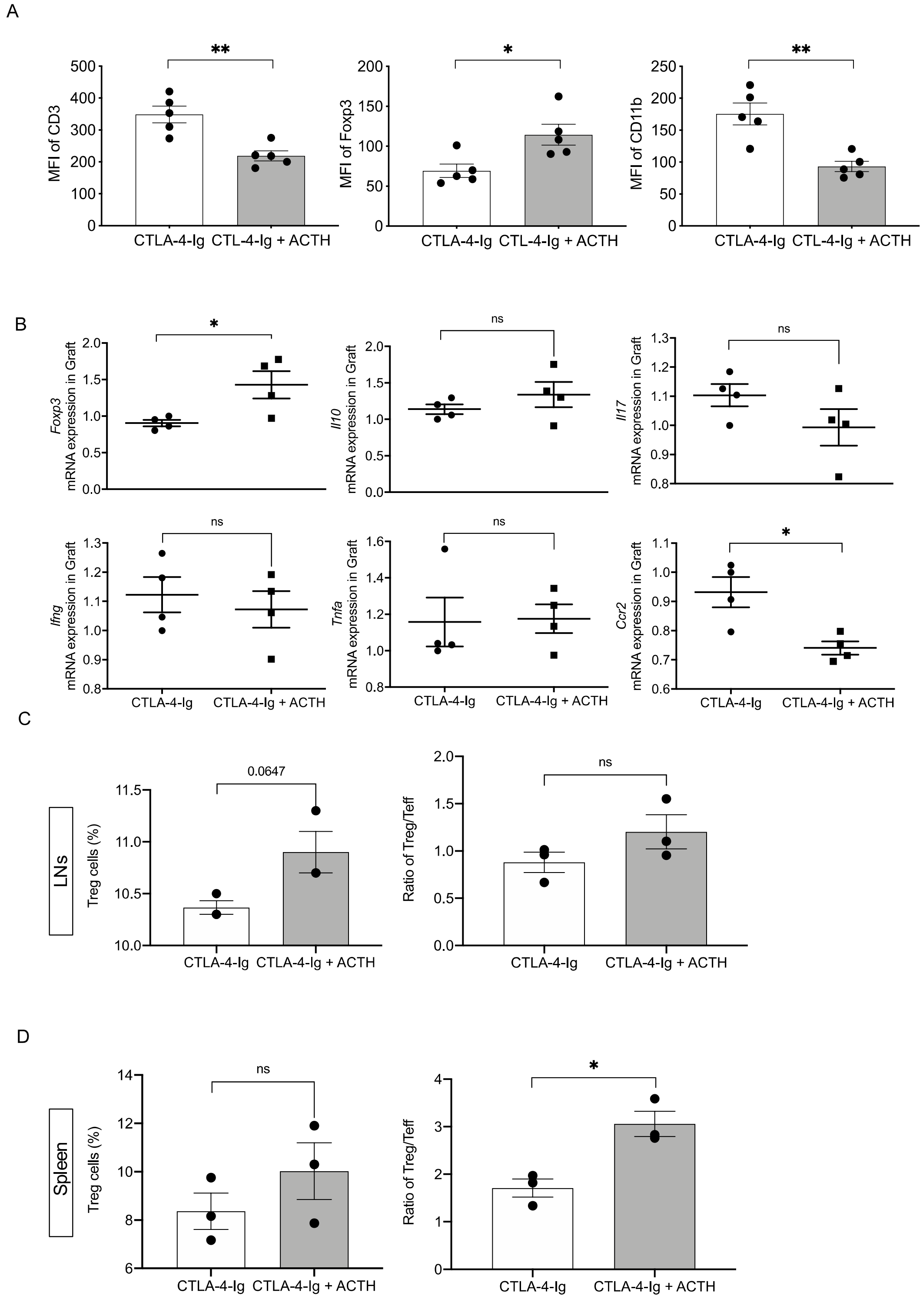

Supplemental Figure 3. (A) Semi-quantitative analysis by MFI of florescence micrographs of heart allograft sections revealed fewer CD3$^+$ T cells and CD11b$^+$ cells, but more Foxp3$^+$ Tregs in group treated with CTLA-4-Ig + ACTH in comparison to group treated with CTLA-4-Ig alone (n=3

allograft/group) (B) qPCR of heart allografts showed significantly higher expression of *Foxp3* and *Ccr2* genes in CTLA-4-Ig + ACTH group, as compared to group treated with CTLA-4-Ig alone. No significant difference in gene expression of *Il10*, *Ifng*, *Tnfa* and *Il17* was observed (n=4 mice/group). (C) Flow cytometric analysis of LNs collected from C57BL/6 recipients at Day 28 showed no difference in the percentages of Tregs and the Treg/Teff ratio between the CTLA-4-Ig group and CTLA-4-Ig + ACTH group. (D) Flow cytometric analysis of spleens collected from C57BL/6 recipients at Day 28 showed no difference in the percentages of Tregs, but significantly higher Treg/Teff ratio was observed in the CTLA-4-Ig + ACTH group in comparison to CTLA-4-Ig group (n=3 mice/group). Data presented as mean ± SEM, * p<0.05, ** P<0.01.

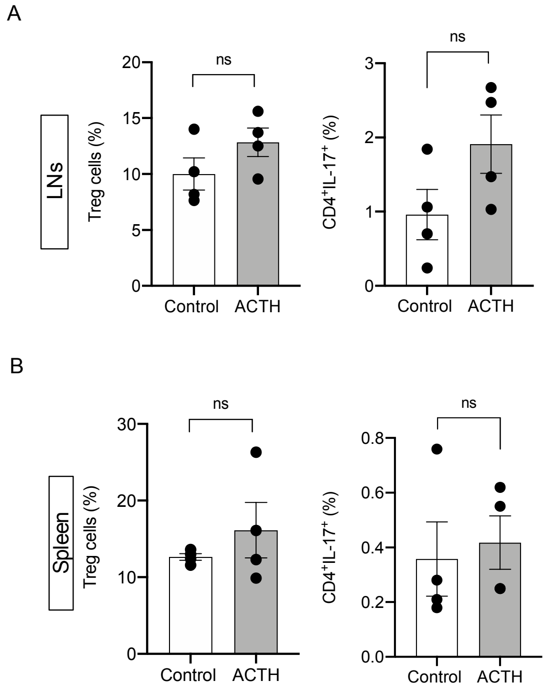

Supplemental Figure 4. (A) Flow cytometric analysis of LNs collected from C57BL/6 recipients at 4 weeks shows no significant difference in the percentages of Tregs between ACTH-treated group and untreated control group. (B) Flow cytometric analysis of spleens collected from

C57BL/6 recipients at 4 weeks shows no significant difference in the percentages of Tregs and $CD4^+IL\text{-}17^+$ cells between the ACTH-treated group and the untreated control group. (n=4 mice/group) Data presented as the mean ± SEM.
